# The influence of experience marketing, sense of virtual community, and satisfaction on user purchase intention in live stream sales

**DOI:** 10.1371/journal.pone.0334231

**Published:** 2025-12-03

**Authors:** Yu-Liang Feng, Ya-Qin You, Shing-Sheng Guan, Zhen-Ning Yuan, Xiao-Yue Chen

**Affiliations:** 1 Artificial Intelligence Innovation Design Research Center, Jiangsu University of Technology, Jiang Su, China; 2 Faculty of Innovation and Design, City University of Macau, Macau; 3 Cross-Strait Vocational Education Integration Development Center, Fujian Information Vocational and Technical College, Fu Jian, China; Westminster International University in Tashkent, UZBEKISTAN

## Abstract

Amid the digital and mobile internet boom, Live Stream Sales has emerged as a transformative, contactless commerce model reshaping consumer behavior. This study examines how diverse user experiences influence Sense of Virtual Community, Satisfaction, and Purchase Intention. The results indicate that think, act, and relate experiences significantly enhance satisfaction; feel and act experiences directly promote purchase intention; and both sense of virtual community and satisfaction serve as key mediators in driving purchase intention. These findings deepen the understanding of user experience in live commerce and provide practical guidance for enhancing consumer engagement and purchase motivation.

## Introduction

Driven by digitalization and mobile internet technology, live-streaming sales have emerged as a significant trend in the e-commerce industry [1]. Starting with Li Jiaqi, a pioneer of live-streaming sales in China, an increasing number of social media influencers, celebrities, entrepreneurs, and grassroots salespeople familiar with the products have joined this movement [2]. Various live-streaming events have not only covered product promotions across regions but successfully integrated traditional sales methods with modern technology, continually expanding its influence.

Live-streaming sales have not only broken the constraints of traditional retail but also crafted a novel shopping experience for consumers through online interaction, personalized service, and real-time demonstration. This experience not only connects users with products but allows them to feel, think, and act within the experience, leaving pleasant memories and thus enhancing the value of the product [1]. Indeed, a positive shopping experience has transcended the influence of price and product, becoming a key determinant in user purchasing behavior, and forging a closer connection between brands and customers [3]. Recent studies have shown that consumers’ purchase intentions can be significantly influenced by how businesses respond to online user reviews, particularly in service contexts like hotels and tourism [4]. Building on this emphasis on enhanced consumer experiences, live e-commerce in China has grown rapidly in recent years, with major e-commerce platforms, video platforms, and enterprises entering the field, driving consumption growth and changing consumer shopping behavior [5,6]. Moreover, the application of multimedia live streaming technology has further enriched the shopping experience, with platform visibility, authenticity, and interactivity significantly influencing consumers’ purchase intentions, thereby promoting the widespread adoption of live commerce [6].

In fact, contemplating from the user’s perspective and employing various experiential marketing techniques have become key factors in the success of live-streaming sales in retaining the current customer base and enhancing customer satisfaction. Experiential marketing, through the creation of rich, immersive shopping experiences, caters to the personalized needs and expectations of consumers, thereby enhancing their purchasing intent and satisfaction. In similar digital commerce scenarios such as cloud kitchens, factors like social media reviews and product knowledge have also been found to shape consumers’ purchase behaviors [7], yet such variables have rarely been systematically integrated into the live-stream commerce context.

Moreover, the sense of virtual community plays a role in consumption behavior and experience as well. By enhancing brand loyalty, fostering interactive communication, and deepening the understanding of products or services, the sense of virtual community can provide consumers with a richer shopping experience. Consumers often interact with others in virtual communities, sharing shopping feelings or seeking purchase advice, with these interactive experiences influenced by the sense of virtual community [8,9,10,11]. Furthermore, in sectors such as boutique hotels, social media marketing efforts that leverage perceived trust and electronic word of mouth have been shown to boost customer purchase intention [12], suggesting that social engagement factors are critical in online service decision-making.

The interaction between experiential marketing and the sense of virtual community plays a crucial role in shaping consumer behavior and satisfaction. However, this relationship remains underexplored in the context of live stream sales. To enrich research in this area, the present study adopts the Stimulus–Organism–Response (SOR) framework, where the five experiential marketing dimensions serve as environmental stimuli, the sense of virtual community and satisfaction represent internal states (organisms), and purchase intention functions as the behavioral response. This model aims to examine how experiential marketing influences purchase intention in live-streaming commerce and to investigate the mediating roles of virtual community engagement and user satisfaction.

### Literature review

This study aims to explore key dimensions such as User Experience (UX), Experience Marketing, User Satisfaction, and Purchase Intention within the context of live-streaming sales. Drawing upon existing theories and models, such as Stimulus-Organism-Response Theory, the study aims to offer insights into the interplay of these variables and their impact on customer purchase decisions.

### User Experience (UE)

User Experience (abbreviated as UE/UX) was proposed by [13]. Hassenzahl [14] believes that user experience is a state of the user’s inner feelings that arise under a specific interaction environment. Park et al. [15] pointed out that user experience includes emotions and user value, mainly including self-satisfaction, pleasure, personalized needs, social representation, and additional meaning of products.

However, the user experience is still a concept that is not clearly defined, and lacks a complete evaluation method and indicators [16,17]. Scholars often divide the dimensions of the user experience based on the characteristics of their research objects. After conducting a survey in the catering industry, Fan and Li [18] divided the dimensions of the catering customer experience into functional experience, emotional experience, and social experience. Pentina et al. [19] identified through exploratory factor analysis that online user experience can be divided into sensory experience, cognitive experience, practical experience, and related experience. Song [20] divided the functional experience in B2C customer experience into website experience, product experience, and service experience through in-depth interviews. Cui and Qu [21] divided the user experience into five dimensions – sense experience, practical experience, product experience, service experience, and relate experience, based on the characteristics of clothing consumers in the cross-border e-commerce online shopping environment. Wu et al. [22] divided the subjective feelings of the restaurant APP interface into emotional experience and functional experience. Previous researchers have conducted in-depth and extensive studies on the influence of online and offline aspects of user experience.

### Experience marketing

Experiential marketing, first proposed by Bernd Schmitt [23], differs from traditional marketing by viewing users as both rational and emotional, emphasizing pleasurable experiences rather than solely product functions and benefits. Its conceptual framework includes Strategic Experiential Modules (SEMs) and Experiential Providers (ExPros). SEMs aim to create distinct user experiences through five types: sense, feel, think, act, and relate experiences. ExPros are strategic tools, such as communication, product presentation, co-branding, environment, website, and personnel, designed to enhance user experience, product value, satisfaction, and loyalty [24]. This study adopts Schmitt’s SEMs classification to measure users’ overall experience in live stream sales.

### Sense of virtual community

The concept of the Sense of Virtual Community (SOVC) has garnered significant attention in research on online communities, especially with the proliferation of digital platforms and social networks. SOVC refers to the feeling of belonging, shared identity, and mutual support among members within a virtual community [25]. Rooted in the psychological sense of community [26], SOVC extends this framework to digital environments, emphasizing elements such as emotional connection, influence, fulfillment of needs, and shared history.

Studies have shown that SOVC plays a critical role in fostering user engagement, satisfaction, and loyalty in online contexts [27]. For instance, Blanchard and Markus [25] highlighted the importance of regular interaction and active participation in building a strong sense of community in virtual spaces. Furthermore, SOVC has been linked to increased trust and cooperative behavior within virtual communities [28]. Factors such as shared goals, effective communication, and emotional support are identified as key antecedents of SOVC [27]. Recent research has also explored the application of SOVC in commercial contexts, such as e-commerce and live stream sales, where it enhances customer engagement and purchase intentions.

Despite its relevance, challenges in measuring SOVC persist due to variations in community structures and user experiences [29]. Future research is needed to address these methodological concerns and to explore SOVC’s dynamics in emerging virtual spaces such as the metaverse. In contemporary platforms like TikTok Live and Twitch, SOVC can be influenced by features such as real-time chat interaction, follower communities, and gamified engagement mechanics. Additionally, new indicators, including live interaction frequency, content co-creation participation, and peer-to-peer support metrics, are emerging to better capture users’ sense of belonging and involvement in live-streaming environments.

### User satisfaction

In the field of product marketing, user satisfaction has always been a focus of attention, used by researchers and practitioners to measure user attitudes towards products. The psychological reflection of the degree of difference between users’ expectations of products (or services) and actual perceptions. When users’ actual perceptions exceed expectations, a state of satisfaction is generated; when actual perceptions do not meet expectations, a state of dissatisfaction is generated. User satisfaction is influenced by user expectations, perceived value, and perceived quality. Perceived value is influenced by user expectations and perceived quality, and perceived quality is directly influenced by user expectations [30].

The research conducted by Ishaq et al. [31] shows that the mediating variables of perceived quality, corporate image, and customer value with customer loyalty are customer satisfaction. Domfeh et al. [32] also saw the mediating role of satisfaction in their research. The study showed that customer satisfaction can mediate the impact of celebrity advertising and celebrity personality on purchase intention. Natalia and Suparna [33] found that customer satisfaction partially mediates the effect of product quality on repurchase intentions. This study will explore user satisfaction as an intermediary variable between user experience and purchase intention, in order to better understand the relationship between user experience and purchase intention.

### Purchase intention

Purchase intention refers to the action tendency of the user towards the product [34], purchase intention is a subjective attitude, and also serves as an important indicator for predicting user behavior [35]. Spears and Singh [36] believe that when users have a good impression or attitude towards a certain product, they may form a purchase intention.

### Stimulus-organism-response theory

Mehrabian and Russell [37] proposed the Stimulus-Organism-Response (SOR) theory to explain how individuals respond to environmental stimuli. The model posits an interactive relationship between stimuli (S), an individual’s physiological and psychological states (O), and behavioral responses (R) [38,39]. S refers to environmental factors; O encompasses prior experiences, beliefs, attitudes, motivations, and cognitive structures; R reflects approach or avoidance behaviors. The SOR framework has been widely applied to understand user behavior in various contexts, including avoidance behavior [40] and online purchasing [41]. In this study, experiential marketing serves as the stimulus, sense of virtual community and satisfaction represent the organism, and user purchase intention reflects the response, aiming to examine the impact of live-streaming marketing experiences on customer purchase intentions.

### Research hypothesis

In the dynamic landscape of live-stream sales, deciphering the intricate factors that shape consumer behavior and purchasing decisions is more critical than ever. This portion of our research adopts a meticulous empirical methodology to scrutinize the interplay between Experience Marketing and key variables such as Sense of Virtual Community, User Satisfaction, and Purchase Intention. We leverage the well-regarded Stimulus-Organism-Response (SOR) theory as our foundational framework, casting the user’s live-stream experience as the ‘Stimulus,’ their emotional engagement and satisfaction levels as the ‘Organism,’ and their eventual purchasing choices as the ‘Response.’ Through an organized sequence of hypotheses, this section aspires to construct a scholarly framework that elucidates the multifaceted impact of live-streaming on consumer behavior.

### Experience marketing and sense of virtual community

When consumers interact with products in a virtual environment, it can stimulate the formation of virtual product experience [42]. Research has found a positive correlation between virtual product experience and sense of virtual community [11]. In the scenario of Live Stream Sales, the sensory experience, emotional experience, thinking experience, action experience, and relationship experience formed by consumers interacting with the host and other consumers in the Live Stream Sales platform constitute a kind of virtual product experience. Therefore, the following hypotheses are proposed:

H1a: Sense experience has a significant positive impact on the sense of virtual community in live stream sales;

H1b: Feel experience has a significant positive impact on the sense of virtual community in live stream sales;

H1c: Think experience has a significant positive impact on the sense of virtual community in live stream sales;

H1d: Act experience has a significant positive impact on the sense of virtual community in live stream sales;

H1e: Relate experience has a significant positive impact on the sense of virtual community in live stream sales;

### Experience marketing and user satisfaction

Experience marketing allows consumers to experience the efficacy of the product and stimulates positive responses [24]. The emotional experience in experience marketing has a significant positive impact on satisfaction [43]. According to a study by Ali et al. [44] on the interaction between tourists’ experience and emotional needs in Malaysian theme parks, there is a significant positive relationship between the level of experience and satisfaction. Based on this, the following hypotheses are proposed:

H2a: Sense experience has a significant positive impact on user satisfaction in live stream sales;

H2b: Feel experience has a significant positive impact on user satisfaction in live stream sales;

H2c: Think experience has a significant positive impact on user satisfaction in live stream sales;

H2d: Act experience has a significant positive impact on user satisfaction in live stream sales;

H2e: Relate experience has a significant positive impact on user satisfaction in live stream sales;

### Experience marketing and purchase intention

Song and Xiao [45] found that the five dimensions of experience have a significant positive impact on users’ purchase intentions when studying the impact of experience marketing on user purchase intention in the telecommunications industry. Hsu and Lü [46] found that the five facets of experience marketing have a significant positive impact on repurchase intention when studying the relationship between experience marketing and the repurchase intention of iPhone users. Chu and Liu [47], using C-Bike as an example, believe that the sensory, emotional, thinking, and action responses that consumers generate during the experience process will prompt consumers to purchase. Prior studies have highlighted that relate experience, which fosters personal identification and social bonding, plays a crucial role in enhancing consumer purchase intention ([24]; Brakus et al., 2009). In the context of live-stream sales, where community engagement and influencer interaction are key, this relational aspect becomes even more influential.Therefore, the following hypotheses are proposed:

H3a: Sense experience has a significant positive impact on user purchase intention in live stream sales;

H3b: Feel experience has a significant positive impact on user purchase intention in live stream sales;

H3c: Think experience has a significant positive impact on user purchase intention in live stream sales;

H3d: Act experience has a significant positive impact on user purchase intention in live stream sales;

H3e: Relate experience has a significant positive impact on user purchase intention in live stream sales;

### Sense of virtual community and user satisfaction, purchase intention

Chen [48] found that sense of virtual community has a positive impact on user usage intention in his study of user behavior in social commerce. Zhen [49] believes that the sense of virtual community has a positive impact on the collective behavior intention of online Q and A community users. Huang [50] found in a study on the influence of perceived factors of travel virtual community users on customer fit attitude, that sense of virtual community has a positive effect on customer fit attitude and indirectly affects fit behavior through fit attitude. Tang [51] found in a study on “Perception of Virtual Community” in Yi Cloud Music, that the three dimensions of the sense of virtual community have a significant positive impact on user purchase intention. Based on this, the following hypotheses are proposed:

H4a: There is a significant positive correlation between the sense of virtual community and user experience satisfaction in live stream sales;

H4b: There is a significant positive correlation between the sense of virtual community and user purchase intention in live stream sales;

### Satisfaction and user purchase intention

Mittal and Kamakura [52] pointed out that only when customers are very satisfied with a product can they be motivated to make repeat purchases. Wang and Liu [53] confirmed in her study that improving user satisfaction in live stream sales can promote user purchase intention, thereby improving sales performance. Li [2] and Li et al. [54] found in the field of mobile commerce that the satisfaction during the user experience process has a significant impact on the intention to continue to use. Song, Zhang ang Hu [55], in his study on restaurant brand equity and user satisfaction, found that there is a significant positive relationship between customer satisfaction and customer conversion intention [56]. A study on online shopping of fresh produce found that improving satisfaction can significantly enhance the intention of users to repurchase [57]. Based on this, the following hypothesis is proposed:

H5: There is a positive correlation between satisfaction and user purchase intention in live stream sales.

### Research model

**Fig 1:**
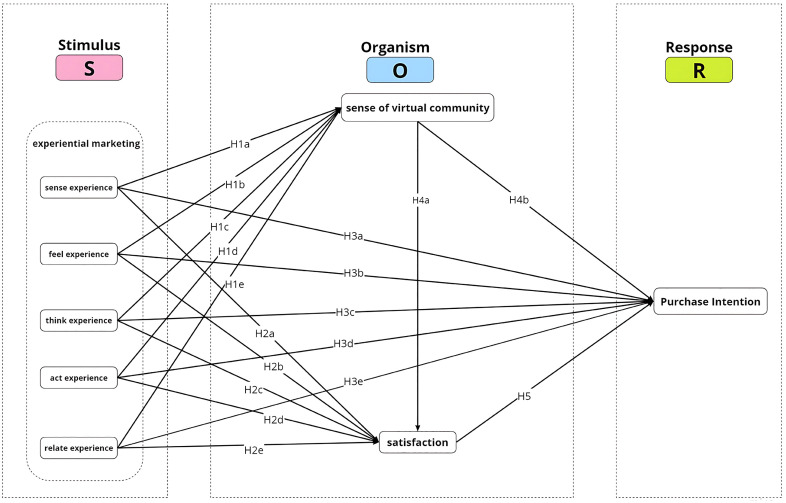
The proposed research model.

The research framework of this study is based on the SOR theory, with user experience marketing projects as stimuli, sense of virtual community and satisfaction as organisms, and purchase intention as the result of behavioral response [58]. The specific illustration is as follows (See Fig 1).

### Methodology

The primary data for this study comes from questionnaire surveys, with sample collection being implemented. Ultimately, the data was analyzed using SPSS 20 and Smart PLS 3 statistical software.

### Measurements of variables

The Live Stream Sales questionnaire is a comprehensive survey compiled from past research results, specifically tailored towards the unique characteristics of live stream sales experience (see Table 1 for example), and has been modified by marketing experts to serve the purpose of this research. The questionnaire consists of 36 items and is divided into two parts: the first part pertains to basic information such as the respondent’s gender, age, occupation, involvement in Live Stream Sales, usage time and platform; the second part deals with experience marketing, purchase intention, satisfaction, and sense of virtual community. Each of the 36 items was rated on a Likert 5-point scale, ranging from strongly disagree (1 point) to strongly agree (5 points).

**Table 1:** Questionnaire setting.

Construct	Dimension	Conceptual definition	Question
First part: experience marketing	Senses experience (SE)has 6 Questions	The experiential context customers come into contact with can affect their cognition and evaluation of products through multiple sensory stimuli such as vision, hearing, touch, taste, and smell, and the sensory impact model can achieve the goals of sensory marketing [59,60,61,62,63].	live stream sales video screen is very clear. The voice of the live stream sales anchor is contagious. live stream sales platform interface is simple and elegant. live stream sales platform interface is sensitive to operation. The image of the anchor in live stream sales is pleasing to the eye or simple and generous. live stream sales product display (method, scale) is appropriate.
	Feel experience(FE) has 5 questions	By providing certain experiences, businesses can evoke users’ emotions towards the brand, which is a strategy primarily emphasizing users’ inner feelings and emotions. The goal is to create emotional experiences, stimulate user emotions, and thus encourage user engagement [59,60,61]; Muhammadet al., 2014; [63].	Participating in live stream sales makes me feel good. Participating in live stream sales relaxes my mind and body. Participating in live stream sales is a pleasure. Participating in live stream sales made me immerse myself in it and forget about my troubles. The price and quality of the products purchased by participating in live stream sales are satisfactory.
	Think experience(TE) has 4 questions	Creative marketing aims to help customers experience, perceive, and solve problems in creative ways by provoking their surprise, delight, and interest, and stimulating intellectual engagement for divergent and convergent thinking [24,64,65].	Participating in live stream sales fills me with curiosity. Participating in live stream sales often surprises me (e.g.,: grabbing a favorite product). Participating in live stream sales gave me the idea of trying to download other live stream sales platforms. Participating in live stream sales always makes me think new.
	Act experience(AE) has 4 Questions	Customer experience involves various aspects such as bodily experience, lifestyle, and social interaction. Mobile marketing influences customers’ lifestyles, enhances the quality of life by augmenting bodily experiences, facilitating social interaction, and promoting new social norms [66]; Gentileet al., 2007; [63,62,24].	live stream sales have changed my lifestyle. Participating in live stream sales has become a part of my life. Watching live stream sales has become an important way for me to relax. live stream sales has changed my shopping style or shopping habits.
	Related experience (RE)has 3 Questions	Self-improvement is a key appeal to win the favor of others and establish close brand relationships and brand communities with others, social culture, and the environment, in order to construct a powerful brand image. The association encompasses senses, emotions, thoughts, and actions [24,64,65].	I feel that participating in live stream sales can give me a sense of belonging to a group. I feel that participating in live stream sales can enhance my connections with others. I feel that participating in live stream sales can help me establish interactive relationships with others.
The second part: Satisfaction and purchase intention	Satisfaction (SAT)has 3 questions	Satisfaction primarily refers to the users’ satisfaction level with the technology, transaction experience, and overall experience on the live stream sales platform [67].	I am quite satisfied with the technology of the live stream sales platform. I am satisfied with the shopping experience at live stream sales. Overall, I am satisfied with the live stream sales platform.
	Purchase Intent (PI)has 2 Questions	Eagly and Chaiken argue that intention has a distinct personal motive and is a conscious effort made by an individual to achieve certain behavior. Other studies have shown that intention is the probability of an individual adopting certain behavior [67].	I often buy favorite items in live stream sales. I will continue to use the live stream sales platform in the future.
The third part: Sense of Virtual Community	Sense of Virtual Community (SoVC)has 9 Questions	Virtual community feeling is the social members’ perception of membership, influence, and immersion in the virtual society. Membership reflects the members’ sense of belonging to the virtual community. Influence reflects the degree of influence on other members within the community. Immersion describes a state of member engrossment in the community, portraying a degree of community involvement (Peng and Shen, 2016.; [68].	I feel like a member of this live stream sales chat room. I feel like the members in the live stream sales chat room are like my good friends. I prefer to communicate with the members of my live stream sales chat room. I often speak in the live stream sales chat room. Many people in the live stream sales chat room responded to my statement. I am an active member in the live stream sales chat room. I will spend a lot of time on live stream sales chat. My participation in live stream sales affected other event arrangements. I often respond positively to other members of the live stream sales chat room.

To ensure data quality, the questionnaire platform employed a forced-response mechanism, preventing item-level missing values. In addition, outlier detection was conducted using boxplot analysis, and responses with extreme values or inconsistent patterns were removed to maintain the stability of the data.

All participants were informed of the study’s purpose, data usage, and anonymity protocols before commencing the survey. Informed consent was obtained electronically at the beginning of the questionnaire through a mandatory agreement checkbox. For participants under the age of 18 (accounting for less than 1% of the sample), additional confirmation was provided that they had obtained the consent of their parents or legal guardians. No personally identifiable information was collected at any stage, and the dataset was securely stored and used solely for academic purposes.

### Sample collection

This research was conducted from January 6, 2022, to March 16, 2022, using the Questionnaire Star platform for online random sampling. In total, 365 volunteers were recruited. To ensure the privacy of the respondents and to eliminate their concerns about answering, an explanation was provided on the first page of the questionnaire, guaranteeing that the data would only be used for academic research and personal information would not be disclosed. In the end, 365 samples were collected. After eliminating 5 samples from those who had never watched Live Stream Sales, a total of 360 valid questionnaires remained.

### Analytical method

This research used SPSS 20 and Smart PLS 3 statistical software for analysis. Descriptive statistical analysis, reliability and validity analysis, confirmatory factor analysis, and structural model analysis were carried out based on the research purpose and hypotheses.

### Smart PLS model specifications and analysis

The research used Smart PLS 3 to implement partial least squares structural equation modeling (PLS-SEM) [69, 70]; Ringleet al., 2015), and used the software for statistical confirmatory factor analysis. PLS-SEM (Partial Least Squares Structural Equation Modeling) is a statistical method used to analyze causal relationships among multiple variables while simultaneously assessing the reliability and validity of measurement models. It is well-suited for studies with small sample sizes, complex models, or non-normally distributed data. Compared to traditional SEM, PLS-SEM focuses more on prediction accuracy and variance explanation, making it widely applied in empirical research in fields like marketing and management [71].This method is suitable for non-normally distributed survey data, and can be applied to small sample sizes and more complex models [72]. As the measurement items were acquired through perceptual scoring, the data were often non-normally distributed [73]. According to Lee et al. [74], the data in this research are non-normally distributed since the p-values of the Kolmogorov-Smirnov normality test were all less than 0.05, thus PLS-SEM was an appropriate analysis method.

### Evaluation of measurement model

During the evaluation of the measurement model, it is necessary to ensure reliability and validity [75]. According to Hair et al. [76], the validity of the measurement items is tested by factor loadings, and whether an item should be removed is judged based on individual factor loadings (>0.3). The results can demonstrate the content validity of the measurement model. Hew et al. [77] proposed that internal consistency reliability can be assessed by two indicators: Cronbach’s alpha (C-α value) >0.7 and composite reliability (CR value) >0.6. When the C-α value is < 0.7, to ensure research content consistency, it is recommended that the item should be removed [78]. According to [76,79], to ensure convergent validity, the average variance extracted (AVE) should be > 0.5 and indicator loading values >0.7. Hew, Badaruddin, and Moorthy [79] proposed the standard for judging discriminant validity, according to which comparison should be made between the diagonal elements. The diagonal elements represent the square root of AVE, and the related values serve as non-diagonal elements. If all diagonal values are higher than the diagonal values in the same row and column, discriminant validity can be inferred [80]. According to Henseler et al. [81], the heterotrait-monotrait ratio of correlations (HTMT) serves as a new standard for assessing discriminant validity, its value should be less than 1 to distinguish one construct from another. Based on this standard, discriminant validity can be further ensured.

### Structural model suitability judgment

Hair et al. [82] argued that in the analysis of the structural model (inner model), a VIF < 10 is appropriate, and anything above 10 is inappropriate. In the judgment of the structural equation model (inner model), two important indicators of the structural model’s judgment are: 1) R square must be > 0.1; 2) Standardized regression coefficient T values must be significant. Generally, non-parametric resampling techniques are used to examine the stability of the bootstrap method evaluated by PLS [83,84].

## Results

Based on statistical data analysis, this study offers a demographic overview of the survey respondents. The questionnaire data is rigorously analyzed across seven key metrics: missing values (capped at 5%), mean, standard deviation, skewness, T-tests, correlations, and factor loadings. Finally, the study assesses the goodness-of-fit of the measurement model and the precision of predictive outcomes.

### Demographic profile of respondents

Based on the analysis of sample background data using SPSS 20 statistical software, the population variable analysis of this study is as follows (Table 2). It should be noted that the sample is skewed toward students (66.1%), which may limit the generalizability of the findings.

**Table 2:** Demographic profile of responde.

Demographic variables	Variable	Frequency allocation	Percentage(%)
Gender	male	137	38.1
	Female	223	61.9
Age	under 18	1	0.3
	18 ~ 25	242	67.2
	26 ~ 30	48	13.3
	31 ~ 40	38	10.6
	41 ~ 50	26	7.2
	51 ~ 60	3	0.8
	over 60	2	0.6
Profession	student	238	66.1
	individual personnel	13	3.6
	salesperson	5	1.4
	public servant	3	0.8
	business unit personnel	53	14.7
	professionals	7	1.9
	other	41	11.4
Which live stream sales APP is the most	Tik Tok	211	58.6
	Kuaishou	11	3.1
	Taobao Live	91	25.3
	JD Video	2	0.6
	Xiaohongshu	6	1.7
	other	5	1.4

### Evaluation of the measurement model

The questionnaire in this study focuses on the issues of user experience in live stream sales. Using SPSS 20 statistical software to analyze the questionnaire data, the seven major indicators including missing values (5%), mean, standard deviation, skewness, T-test, correlation, and factor loadings were all found to be within reasonable ranges (Table 3). Common method bias was assessed via Harman’s single-factor test, with the variance explained by the first factor well below the 40% threshold, indicating minimal bias.

**Table 3:** Seven indicators test.

Serial number	Mean	Standard deviation	Skewness	Extreme group t-test	Related a	Factor loading
Q1	3.78	0.93	−0.503	8.226***	0.512	0.519
Q2	3.68	0.966	−0.285	9.713***	0.572	0.581
Q3	3.45	1.015	−0.218	14.077***	0.671	0.677
Q4	3.58	0.949	−0.081	9.673***	0.57	0.577
Q5	3.47	0.959	−0.123	11.625***	0.660	0.668
Q6	3.63	0.864	−0.113	9.093***	0.566	0.576
Q7	3.31	0.991	−0.075	14.855***	0.767	0.777
Q8	3.25	0.977	−0.018	17.271***	0.803	0.812
Q9	3.15	1.019	0.065	16.614***	0.807	0.817
Q10	3.04	1.047	0.173	17.944***	0.809	0.816
Q11	3.33	0.924	−0.034	13.637***	0.706	0.712
Q12	3.39	0.95	−0.102	13.482***	0.741	0.749
Q13	3.43	0.966	−0.265	14.424***	0.755	0.765
Q14	2.92	1.108	0.100	16.49***	0.766	0.771
Q15	3.14	0.998	0.102	14.793***	0.786	0.79
Q16	3.02	1.063	−0.022	15.683***	0.758	0.764
Q17	2.72	1.151	0.164	20.037***	0.815	0.821
Q18	2.87	1.153	0.031	20.656***	0.842	0.848
Q19	2.96	1.114	−0.078	18.465***	0.821	0.827
Q20	3.43	0.982	−0.488	12.636***	0.739	0.752
Q21	3.22	0.999	−0.205	15.512***	0.810	0.819
Q22	3.13	0.992	−0.085	17.004***	0.829	0.836
Q23	2.96	1.056	0.023	17.674***	0.837	0.843
Q24	2.76	1.084	0.18	18.725***	0.838	0.845
Q25	2.83	1.099	0.054	17.762***	0.827	0.834
Q26	2.62	1.158	0.363	17.111***	0.799	0.808
Q27	2.80	1.079	0.090	16.969***	0.823	0.829
Q28	2.54	1.163	0.390	17.551***	0.784	0.793
Q29	2.41	1.203	0.473	16.115***	0.770	0.779
Q30	2.47	1.214	0.446	13.751***	0.718	0.725
Q31	2.53	1.172	0.331	16.758***	0.765	0.775
Q32	3.31	0.916	−0.186	12.337***	0.750	0.762
Q33	3.22	0.882	−0.084	13.082***	0.774	0.787
Q34	3.32	0.884	−0.213	12.419***	0.749	0.762
Q35	3.00	1.091	−0.065	16.222***	0.788	0.797
Q36	3.25	0.986	−0.283	14.942***	0.778	0.788

This study uses Smart PLS 3 for Partial Least Squares Structural Equation Modeling (PLS-SEM) [69],Hew et al., 2014; [85], and uses its statistical software for confirmatory factor analysis. This method is suitable for non-normal distribution of survey data and is applicable to small sample sizes and more complex models [72]. Since measurement items are obtained through perceived scoring, it is difficult to prove that the data follows a normal distribution [73]. Based on the recommendation by Lee et al. [74], and Ooi and Tan [75], the Kolmogorov-Smirnov normality test was used to validate the data of this study, and the result was p-value > 0.05, indicating that the data of this study belongs to the type of non-normal distribution and can be analyzed using the PLS-SEM method. Although the number of samples collected in this study is small (360), it still meets the minimum sample size calculation proposed by Soper [86]. Combining the results of the reliability and validity test standards of the PLS-SEM research method (Tables 4–7), questions Q7, Q8, and Q9 were deleted due to their collinearity value exceeding 10, and all other indicators were within the specified range.

**Table 4:** AVE and reliability measures.

Project	AVE value	CR value	C-α value	Factor loading
TE	0.782	0.935	0.907	>0.8
FE	0.857	0.923	0.833	>0.8
SE	0.728	0.941	0.925	>0.8
SAT	0.894	0.962	0.940	>0.8
SoVC	0.765	0.961	0.967	>0.8
AE	0.838	0.954	0.935	>0.8
PI	0.899	0.947	0.888	>0.8
RE	0.864	0.950	0.921	>0.8

1.SE = Senses experience, FE = Feel experience, TE = Think experience, AE = Act experience, RE = Related experience, SAT = Satisfaction, PI = Purchase Intent,SoVC = Sense of Virtual Community.

2.AVE value>0.5; CR > 0.5; C-α value>0.7; factor loading>0.5.

**Table 5:** The discriminant valid.

	TE	FE	SE	SAT	SOVC	AE	PI	RE
TE	0.885							
FE	0.816	0.926						
SE	0.733	0.726	0.853					
SAT	0.760	0.699	0.661	0.945				
SoVC	0.838	0.775	0.656	0.817	0.875			
AE	0.776	0.731	0.595	0.663	0.847	0.915		
PI	0.747	0.731	0.623	0.813	0.817	0.743	0.948	
RE	0.805	0.765	0.718	0.813	0.910	0.776	0.774	0.930

1.SE = Senses experience, FE = Feel experience, TE = Think experience, AE = Act experience, RE = Related experience, SAT = Satisfaction, PI = Purchase Intent,SoVC = Sense of Virtual Community.

**Table 6:** HTMT value.

	TE	FE	SE	SAT	SOVC	AE	PI	RE
TE								
FE	0.937							
SE	0.797	0.824						
SAT	0.822	0.790	0.708					
SOVC	0.896	0.863	0.688	0.856				
AE	0.844	0.826	0.638	0.706	0.893			
PI	0.832	0.850	0.684	0.889	0.884	0.814		
RE	0.880	0.872	0.776	0.873	0.960	0.835	0.856	

1.SE = Senses experience, FE = Feel experience, TE = Think experience, AE = Act experience, RE = Related experience, SAT = Satisfaction, PI = Purchase Intent,SoVC = Sense of Virtual Community.

2.HTMT value < 1.

**Table 7:** Collinearity value VIF.

VIF value	number	Q1	Q10	Q11	Q12	Q13	Q14	Q15	Q16	Q17
value	2.200	2.037	2.037	3.195	3.067	2.485	3.129	2.906	4.600
number	Q18	Q19	Q2	Q20	Q21	Q22	Q23	Q24	Q25
value	3.965	3.872	2.490	3.121	3.774	3.452	3.800	3.404	4.305
number	Q3	Q26	Q27	Q28	Q29	Q30	Q31	Q32	Q33
value	3.142	4.135	4.932	6.066	6.724	5.601	5.821	4.578	4.747
number	Q34	Q35	Q4	Q36	Q3	Q5	Q6		
value	3.828	2.760	2.746	2.760	2.760	3.380	2.703		

VIF value <10.

In previous research, it was found that the Goodness of Fit (GOF) is an important model fit indicator for the Partial Least Squares method structural equation model. When GOF > 0.36, it indicates that the model has a high degree of fit; when 0.24 > GOF > 0.10, it indicates an acceptable degree of fit; when GOF < 0.10, it indicates a poor model fit [87]. The mathematical calculation formula for GOF is as follows:


$$\text{GOF}=\sqrt{\text{averageAVE}+R^{\text{2}}}$$

The GOF calculation result for this study model was 0.806, indicating that the model, which includes the five factors of experience marketing, Sense of Virtual Community, satisfaction, and purchase intention, has a high degree of fit.

### Examining the structural model and conclusion

To verify the stability of the model, researchers carried out 5000 computations using the Bootstrap method in SmartPLS. According to the research findings of Hair et al. [88], the R² coefficient can accurately assess the predictive accuracy of the model. An R² coefficient > 0.7 indicates a significant level of predictive accuracy; 0.7 > R² coefficient > 0.5 indicates a moderate level of predictive accuracy; 0.5 > R² coefficient > 0.25 indicates a low level of predictive accuracy; and an R² coefficient < 0.2 suggests the predictive level is not significant.

**Fig 2:**
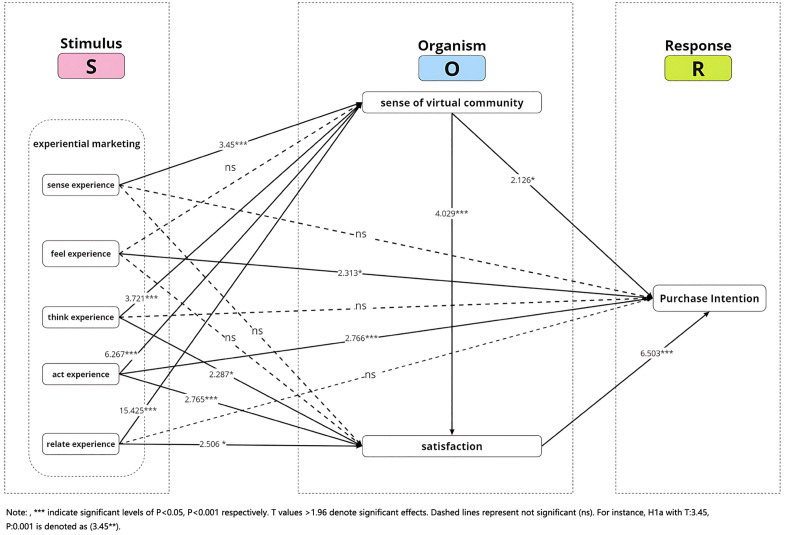
Result of structural model.

The study concludes that for Hypothesis H1a: Sense Experience → Sense of Virtual Community, its T-value is 3.45, P-value < 0.05, indicating a significant positive impact, the hypothesis is verified; for Hypothesis H1b: Feel Experience → Sense of Virtual Community, its T-value is 1.139, P-value > 0.05, indicating a positive but insignificant impact, the hypothesis is not verified. This may suggest that the emotional immersion provided by live-streaming platforms is relatively weaker than that of other online environments, limiting the influence of feel experience on users’ sense of virtual community. Further theoretical exploration is needed to understand contextual factors that may moderate this relationship; for Hypothesis H1c: Think Experience → Sense of Virtual Community, its T-value is 3.721, P-value < 0.05, indicating a significant positive impact, the hypothesis is verified; for Hypothesis H1d: Act Experience → Sense of Virtual Community, its T-value is 6.267, P-value < 0.05, indicating a significant positive impact, the hypothesis is verified; for Hypothesis H1e: Relate Experience → Sense of Virtual Community, its T-value is 15.425, P-value < 0.05, indicating a significant positive impact, the hypothesis is verified;

For Hypothesis H2a: Sense Experience → Satisfaction, its T-value is 1.921, P-value > 0.05, indicating a positive but insignificant impact, the hypothesis is not verified; for Hypothesis H2b: Feel Experience → Satisfaction, its T-value is 0.361, P-value > 0.05, indicating a positive but insignificant impact, the hypothesis is not verified; for Hypothesis H2c: Think Experience → Satisfaction, its T-value is 2.287, P-value < 0.05, indicating a significant positive impact, the hypothesis is verified; for Hypothesis H2d: Act Experience → Satisfaction, its T-value is 2.765, P-value < 0.05, indicating a significant positive impact, the hypothesis is verified; for Hypothesis H2e: Relate Experience → Satisfaction, its T-value is 2.506, P-value < 0.05, indicating a significant positive impact, the hypothesis is verified;

For Hypothesis H3a: Sense Experience → Purchase Intention, its T-value is 0.071, P-value > 0.05, indicating a positive but insignificant impact, the hypothesis is not verified; for Hypothesis H3b: Feel Experience → Purchase Intention, its T-value is 2.313, P-value < 0.05, indicating a significant positive impact, the hypothesis is verified; for Hypothesis H3c: Think Experience → Purchase Intention, its T-value is 0.335, P-value > 0.05, indicating a positive but insignificant impact, the hypothesis is not verified; for Hypothesis H3d: Act Experience → Purchase Intention, its T-value is 2.766, P-value < 0.05, indicating a significant positive impact, the hypothesis is verified; for Hypothesis H3e: Relate Experience → Purchase Intention, its T-value is 0.585, P-value > 0.05, indicating a positive but insignificant impact, the hypothesis is not verified;

For Hypothesis H4a: Sense of Virtual Community → Satisfaction, its T-value is 4.029, P-value < 0.05, indicating a significant positive impact, the hypothesis is verified; for Hypothesis H4b: Sense of Virtual Community → Purchase Intention, its T-value is 2.126, P-value < 0.05, indicating a significant positive impact, the hypothesis is verified;

For Hypothesis H5: Satisfaction → Purchase Intention, its T-value is 6.503, P-value < 0.05, indicating a significant positive impact, the hypothesis is verified (see Table 8 for details and Fig 2).

**Table 8:** Result of structural model.

Hypotheses	Paths	Path coefficients (β)	T-values	P-Values	Remarks
H1a	SE →SoVC	−0.097	3.450	0.001***	Supported
H1b	FE→SoVC	0.046	1.139	0.255	Unsupported
H1c	TE→SoVC	0.199	3.721	0.000***	Supported
H1d	AE→SoVC	0.273	6.267	0.000***	Supported
H1e	RE→SoVC	0.574	15.425	0.000***	Supported
H2a	SE → SAT	0.107	1.921	0.055	Unsupported
H2b	FE → SAT	0.025	0.361	0.718	Unsupported
H2c	TE → SAT	0.170	2.287	0.022	Supported
H2d	AE → SAT	−0.17	2.765	0.006***	Supported
H2e	RE → SAT	0.282	2.506	0.012***	Supported
H3a	SE → PI	0.004	0.071	0.944	Unsupported
H3b	FE → PI	0.154	2.313	0.021***	Supported
H3c	TE → PI	−0.025	0.335	0.738	Unsupported
H3d	AE → PI	0.190	2.766	0.006***	Supported
H3e	RE → PI	−0.055	0.585	0.559	Unsupported
H4a	SoVC → SAT	0.472	4.029	0.000***	Supported
H4b	SoVC → PI	0.246	2.126	0.034***	Supported
H54a	SAT → PI	0.440	6.503	0.000***	Supported

1.SE = Senses experience, FE = Feel experience, TE = Think experience, AE = Act experience, RE = Related experience, SAT = Satisfaction, PI = Purchase Intent,SoVC = Sense of Virtual Community.

2.*** indicate significant levels of P < 0.05.

### Predictive relevance

In the research, in addition to R2 which can predict the accuracy of the model, this study also tested the predictive relevance of the internal structure of the model with Stone-Geisser’s Q2 statistical data.Use Partial Least Squares (PLS) software for analysis, and calculate and output statistical metrics such as R2and Q2。When Q2 > 0.35, the predictive relevance is high; when 0.35 > Q2 > 0.15, the predictive relevance is moderate; when 0.15 > Q2 > 0.05, the predictive relevance is low; when Q2 < 0.05, there is no predictive relevance [89]. In this study, we found from Table 9 that all Q2 statistical data are greater than 0.6, indicating that the endogenous structure of the model can provide high predictive accuracy.In addition to R² and Q², the SRMR value of this study (<0.08) ensured good model fit;

**Table 9:** Predictive accuracy and predictiv.

Project	R^2^	Q^2^
Satisfaction	0.72	0.636
sense of virtual community	0.89	0.669
Purchase Intention	0.75	0.661

### Inspecting the mediating effects

**Fig 3:**
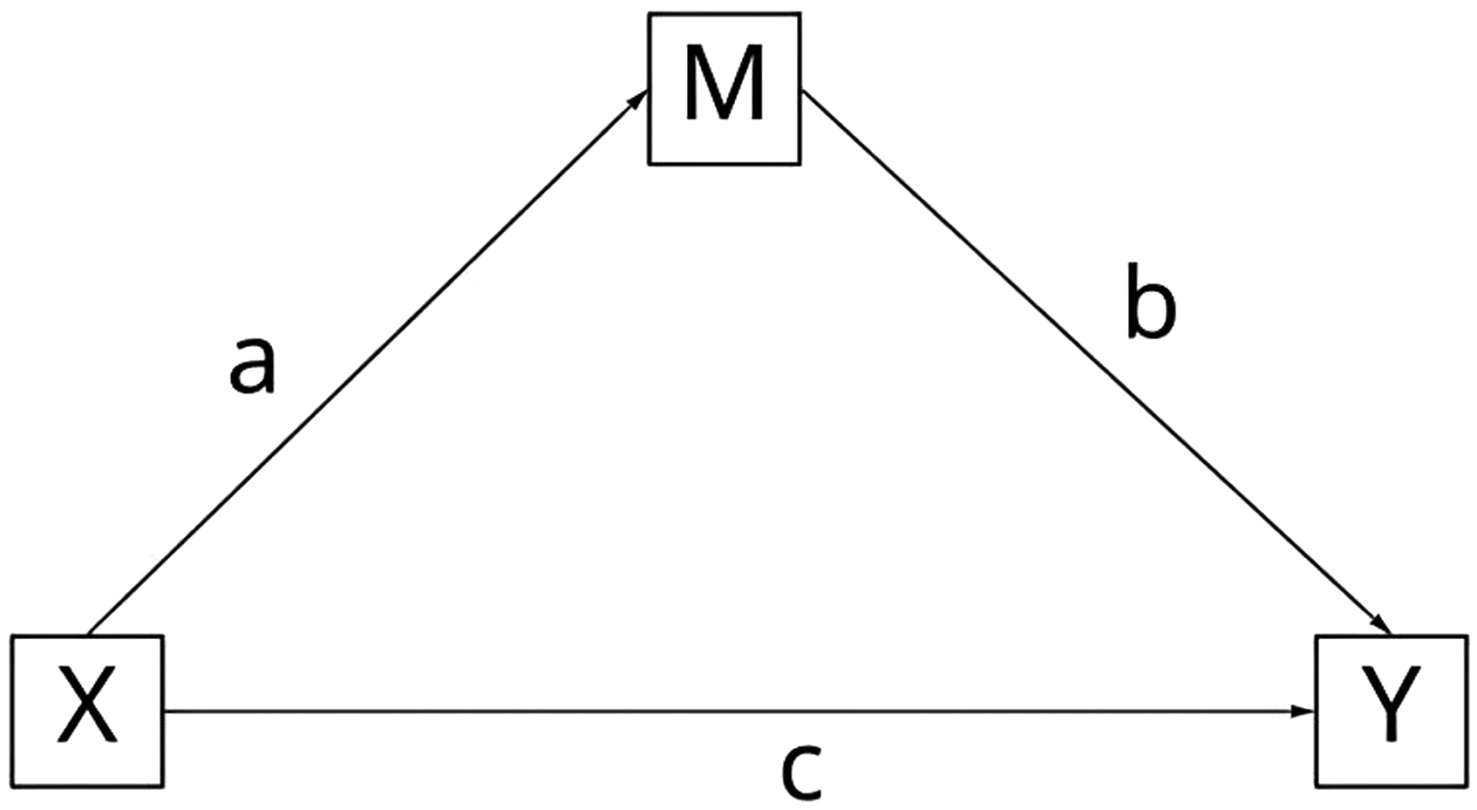
Mediation effect model.

The method of examining the mediating effects in this study was based on the inspection standard proposed by Hair et al. [88]: When the Variance Accounted For (VAF) > 80%, it indicates complete mediation; when 20% < VAF < 80%, it indicates partial mediation; when VAF < 20%, it indicates no mediation. In PLS, researchers will calculate the strength of the mediation. One method is to calculate the ratio of the indirect effect to the total effect(See Fig. 3). This ratio is also known as the Variance Accounted For (VAF) value.

Step 1: Test the significance of the coefficient predicting Y from X (c).

Step 2: Test the significance of the coefficients predicting M from X and Y from M (a and b).

Step 3: Implement the full mediation model, validate the significance of the coefficients (a, b, and c), evaluate the explained variance (VAF), the calculation formula is as follows:


$$VAF=\frac{a\times b}{(a\times b)+c}$$

(Note: a, b, c are path coefficients)

In the results of the mediation effect study, it was found that Satisfaction does not mediate between Influence, Feel Experience and Purchase Intention; Satisfaction partially mediates between Immersion, Think Experience, Relate Experience, Membership and Purchase Intention; Satisfaction shows a complete mediating effect between Think Experience and Act Experience (see Table 10).

**Table 10:** Mediation test.

Project	Direct effect	T value	P Values	indirect effect	T value	P Values	Total effect	VAF	Mediation effect
AE→SoVC → PI	0.190	2.730	0.006	0.067	3.494	0.000	0.257	26.07%	PME
TE→SoVC → SAT	0.170	2.250	0.025	0.094	2.599	0.009	0.264	35.61%	PME
AE→SoVC → SAT	−0.170	2.718	0.007	0.129	2.023	0.043	−0.041	−314.63%	NM
RE→SoVC → SAT	0.282	2.483	0.013	0.271	4.045	0.000	0.553	49.01%	PME
AE → SAT → PI	−0.170	2.718	0.007	−0.075	2.394	0.017	−0.245	30.61%	PME
SoVC → SAT → PI	0.472	4.062	0.000	0.208	3.351	0.001	0.680	30.59%	PME

1.SE = Senses experience, FE = Feel experience, TE = Think experience, AE = Act experience, RE = Related experience, SAT = Satisfaction, PI = Purchase Intent,SoVC = Sense of Virtual Community, PME = Partial mediation effect, NM = no mediation.

### Discussion and suggestions

This study employs the Stimulus-Organism-Response (SOR) theory as the research framework to explore the relationship between experience marketing factors and the sense of virtual community, user satisfaction, and purchase intention in the context of live-stream marketing. Through literature review, questionnaire surveys, and statistical data analysis, the study ultimately provides recommendations and Suggestions.

## Discussion

The study results reveal that during the user experience of Live Stream Sales, sense experience, think experience, act experience, and relate experience have significant direct impacts on the Sense of Virtual Community (respectively H1a, H1c, H1d, H1e), but the impact of feel experience on the Sense of Virtual Community is not significant (H1b). This finding regarding feel experience is inconsistent with previous assumptions, while the other aspects align with previous studies that found the experience model has significant impacts on some elements of the Sense of Virtual Community [53,11].This may reflect a culturally rooted preference for utilitarian consumption in Chinese livestream environments. Users tend to prioritize pragmatic benefits—discounts, product details, or efficiency—over emotional resonance. In livestreams, emotional cues are mostly delivered by the host, while the audience plays a passive role, weakening the salience of emotional experiences.

During the user experience of Live Stream Sales, think experience, act experience, and relate experience have significant direct impacts on Satisfaction (respectively H2c, H2d, H2e), which is consistent with some previous studies [43,20,1]. However, the impacts of sense experience and feel experience on Satisfaction are not significant (H2a, H2b), which is different from previous studies [43,20,1]. During the process of users participating in Live Stream Sales, more thinking factors, act experience factors, and relate experience factors may increase users’ Satisfaction.

Feel experience and act experience have significant direct impacts on Purchase Intention (respectively: H3b, H3d), which is consistent with previous research (Xu and Lü, 2018; [90]). This suggests that the higher the user’s feel experience and act experience in the live streaming process, the stronger the Purchase Intention. The impact of relate experience on Purchase Intention is not significant (H3e), which is consistent with previous research [90]. The impacts of sense experience, think experience, relate experience on Purchase Intention are not significant (H3a, H3c), which is inconsistent with previous research (Xu and Lü, 2018; [90]. This suggests that sense experience, and think experience cannot effectively promote users to increase Purchase Intention during Live Stream Sales.The present study reveals that think experience and senses experience exert no significant influence on users’ purchase intention in the context of live-streaming commerce. This finding may be attributed to the unique characteristics of live-stream shopping, where rapid pacing, high interactivity, and information overload prompt consumers to engage in emotionally driven, heuristic decision-making. As a result, there is limited cognitive elaboration, thereby diminishing the effect of think experience on behavioral outcomes. Similarly, the sensory experience in live-streaming is largely confined to visual and auditory stimuli, with tactile, olfactory, and other embodied dimensions absent. This constrained and largely passive sensory input may enhance initial attention but fails to generate sufficient experiential depth to translate into strong behavioral intentions. These results suggest that, within fast-paced digital sales environments, rational cognition and passive sensory cues may play a secondary role in shaping purchase decisions, compared to more immersive or emotionally resonant experiences.

The research results show that the Sense of Virtual Community has a significant direct impact on the Satisfaction and Purchase Intention of Live Stream Sales users (H4a), which is consistent with the research results of Naranjo-Zolotov et al. [91]. In addition, the Sense of Virtual Community also has a significant impact on users’ Purchase Intention (H4b), which is in line with the research results of Huang, Hsiao and Chen [92]. The research shows that improving users’ Sense of Virtual Community can significantly improve the Satisfaction and Purchase Intention of Live Stream Sales users.

Furthermore, this study also found a significant relationship between the Satisfaction and Purchase Intention of Live Stream Sales users (H5), which is consistent with the research results of Mittal and Kamakura [52], Wang and Liu [53], Li, Gu and Cao [2], Song et al. [55] and Ma et al. [57]. The research shows that improving the Satisfaction of Live Stream Sales users can significantly enhance their Purchase Intention.

Simultaneously, this study also found that Satisfaction has a partial mediating effect between act experience, Sense of Virtual Community, and Purchase Intention. Specifically, the Sense of Virtual Community has a partial mediating effect between think experience, relate experience, and user Satisfaction; there is no mediating effect of the Sense of Virtual Community between act experience and user Satisfaction; between act experience and user Purchase Intention, the Sense of Virtual Community has a partial mediating effect.

### Suggestions

Based on the empirical findings, we propose the following actionable strategies for marketers, platform operators, and live stream content creators to enhance consumer engagement and drive purchase intention:

1. Design Multi-Sensory Touchpoints to Reinforce Community Feelings: Although sensory experience does not directly drive purchases, it plays a pivotal role in enhancing users’ sense of virtual community. Marketers should enrich visual and auditory elements with high-definition video, dynamic graphics, background music, and professional audio. Where possible, AR/VR or haptic feedback technologies can be employed to simulate immersive product interaction, deepening user involvement and laying the foundation for loyalty and conversion. For example, practitioners can integrate 360° product views in live streams, allow virtual try-on of clothing or accessories using AR, or provide tactile feedback through haptic-enabled devices for product demonstrations, offering users a more realistic and engaging experience.
2. Harness Emotional Resonance to Drive Impulse Buying:Emotional engagement is a key driver of purchase intention. Practitioners are advised to embed relatable narratives, humor, empathy, and moments of surprise into content. Creating a warm, relaxed, or entertaining atmosphere fosters emotional intimacy, which not only improves the viewing experience but increases the likelihood of immediate purchasing behavior.
3. Use Educational Hooks to Stimulate Cognitive Involvement:While think experience may not have a direct effect on purchasing, it exerts a positive indirect influence via satisfaction and community bonding. Consider blending “shoppertainment” with infotainment, such as behind-the-scenes product demonstrations, tips, or mini-tutorials. These tactics enhance users’ perceived value and deepen their cognitive engagement, building trust and affinity with the brand.
4. Encourage User Participation to Activate Behavioral Engagement:Act experience—such as interaction, call-to-action tasks, and gamification—has both direct and mediated effects on purchase intention. Marketers can integrate limited-time offers, comment-based raffles, or co-creation sessions (e.g., voting on product designs) to reinforce users’ role as active participants, thereby embedding purchasing into a broader lifestyle ritual.
5. Strengthen Brand-Person Connection to Build Loyalty:Relate experience enhances user satisfaction and community sense. Brands should highlight values, identity cues, or cultural symbols that allow users to identify with the brand persona. Personalized shout-outs, user-generated content, or ambassador programs can deepen symbolic affiliation and transform passive viewers into loyal brand advocates.
6. Cultivate a Strong Virtual Community for Retention and Advocacy:A strong sense of virtual community is a crucial psychological mechanism behind user satisfaction and repeat purchases. Live-streaming platforms should enable real-time interaction (e.g., live chats, “bullet comments”), follower badges, social groups, and after-sale engagement channels. Sustaining user interaction beyond the stream increases retention, nurtures peer influence, and promotes ongoing purchasing behaviors.

### Research WW and prospects

Although this study presents statistically valid and theoretically supported conclusions, several limitations must be acknowledged. First, the sample was primarily drawn from university students and social media users in mainland China, resulting in a relatively young and geographically concentrated respondent group. This may limit the external validity and generalizability of the findings across broader demographic and cultural contexts.

Second, the study adopted a cross-sectional survey design, which restricts the ability to infer causality between variables. While the structural relationships are statistically significant, they only reflect correlations at a single point in time. Future studies could adopt longitudinal or experimental designs to strengthen causal inference.

Third, potential self-selection bias may exist, as individuals who are more engaged with live stream sales are more likely to participate in such surveys. This could result in over representation of high-involvement users, potentially skewing the findings.

In terms of research expansion, future studies are encouraged to conduct cross-cultural validation of the proposed model in different socio-cultural settings, such as Western markets, to examine whether the influence of experiential marketing and virtual community varies across cultures. Researchers may also consider integrating individual psychological variables such as perceived risk, personal innovativeness, or cultural dimensions like collectivism vs. individualism as moderators to enrich the explanatory power of the model.

Moreover, objective physiological or behavioral tracking methods (e.g., eye tracking, EEG) could be introduced in future experiments to complement self-reported data and reduce subjective bias. Finally, the Kano model of quality classification may be employed in future work to assess which dimensions of user experience are deemed essential, attractive, or indifferent by different consumer segments, providing practical insights for targeted marketing strategies.

As a new and increasingly popular form of green commerce, Live Stream Sales offers notable advantages in reducing physical retail costs and resource consumption. With the continued development of digital infrastructure and user behavior evolution, this model is expected to further expand, warranting deeper and broader exploration in future academic research.
